# Ultrasmall Superparamagnetic Iron Oxide Labeled Silk Fibroin/Hydroxyapatite Multifunctional Scaffold Loaded With Bone Marrow-Derived Mesenchymal Stem Cells for Bone Regeneration

**DOI:** 10.3389/fbioe.2020.00697

**Published:** 2020-06-30

**Authors:** Qin Liu, Longbao Feng, Zelong Chen, Yong Lan, Yu Liu, Dan Li, Chenggong Yan, Yikai Xu

**Affiliations:** ^1^Department of Medical Imaging Center, Nanfang Hospital, Southern Medical University, Guangzhou, China; ^2^Key Laboratory of Biomaterials of Guangdong Higher Education Institutes, Guangdong Provincial Engineering and Technological Research Center for Drug Carrier Development, Department of Biomedical Engineering, Jinan University, Guangzhou, China; ^3^Guangzhou Beogene Biotech Co., Ltd., Guangzhou, China

**Keywords:** tissue engineering, multifunctional scaffold, bone marrow-derived mesenchymal stem cells, bone regeneration, magnetic resonance imaging

## Abstract

Numerous tissue-engineered constructs have been investigated as bone scaffolds in regenerative medicine. However, it remains challenging to non-invasively monitor the biodegradation and remodeling of bone grafts after implantation. Herein, silk fibroin/hydroxyapatite scaffolds incorporated with ultrasmall superparamagnetic iron oxide (USPIO) nanoparticles were successfully synthesized, characterized, and implanted subcutaneously into the back of nude mice. The USPIO labeled scaffolds showed good three-dimensional porous structures and mechanical property, thermal stability for bone repair. After loaded with bone marrow-derived mesenchymal stem cells (BMSCs), the multifunctional scaffolds promoted cell adhesion and growth, and facilitated osteogenesis by showing increased levels of alkaline phosphatase activity and up-regulation of osteoblastic genes. Furthermore, *in vivo* quantitative magnetic resonance imaging (MRI) results provided valuable information on scaffolds degradation and bone formation simultaneously, which was further confirmed by computed tomography and histological examination. These findings demonstrated that the incorporation of USPIO into BMSCs-loaded multifunctional scaffold system could be feasible to noninvasively monitor bone regeneration by quantitative MRI. This tissue engineering strategy provides a promising tool for translational application of bone defect repair in clinical scenarios.

## Introduction

Over the past decades, large progress has been made to develop new scaffolds and strategies in the field of bone tissue engineering. Many different bone implant materials have been designed and evaluated in recent years (Bose et al., 2012; Walmsley et al., 2015; Roman et al., 2017; Roseti et al., 2017). However, only very few of the works have been translated into clinical practice successfully on account of various limitations. One of the challenges to be addressed is the lack of effective methods to track the fate and function of these materials upon implantation (Tang et al., 2016; Sajesh et al., 2019). Tissue grafts nowadays is usually multifaceted and may include cells, biomolecules, and biomaterials. Hence, tissue regeneration has proven problematic partly because the healing and remodeling process remains poorly understood. Plenty of tissue engineering studies still utilize conventional tools, such as histological techniques. This requires tissue specimens by invasive methods, meaning that long-term follow-up assessment is extremely limited (Seung et al., 2015). Therefore, strategies for non-invasive imaging show great potential in the field of bone tissue engineering to facilitate longitudinal assessment of implants.

Non-invasive imaging modalities *in vivo* include optical imaging, micro-computed tomography, magnetic resonance imaging (MRI), and positron emission tomography (Appel et al., 2013; Nam et al., 2015; Eisenstein et al., 2016). In particular, MRI is highly suitable for monitoring tissue-engineered implants, owing to its safety without radiation exposure, excellent soft-tissue contrast, and high resolution without penetration depth restriction. However, the application of MRI in tissue engineering is often hampered by the inherent low contrast of the prepared biomaterials. Hence, some recent reports focused on the incorporation of contrast agents in the biomaterials, such as the ultrasmall superparamagnetic iron oxide (USPIO), to make tissue-engineered scaffolds traceable (Singh et al., 2014a; Sun et al., 2014; Mertens et al., 2015; Wang et al., 2017; Hu et al., 2018). Ultrasmall superparamagnetic iron oxide nano-particles can dramatically shorten the transverse relaxation time (T2) with high sensitivity, which opens up new perspectives for tissue engineering. It has been demonstrated that incorporation of USPIO into collagen-based scaffolds can successfully visualize their location and degradation by MRI (Mertens et al., 2014).

Biomaterials play a crucial role in bone tissue engineering by providing a three-dimensional (3D) scaffold to support cell proliferation and deposition of the extracellular matrix (Ho-Shui-Ling et al., 2018). Recently, silk fibroin (SF) received intensive attention as a biomaterial in fabrication of 3D porous scaffolds because of its unique mechanical properties and tunable biodegradation rate (Yang et al., 2007; Kasoju and Bora, 2012; Ma et al., 2018). However, pristine silk has high solubility and hence presents higher degradation rates compared to natural bone. In this sense, hydroxyapatite (HA), the most present mineral in bone, has been widely used to fabricate scaffolds for bone repair (Zhou and Lee, 2011). Recent studies showed that scaffolds composed of different combinations of SF and HA presented great potential for skeletal regeneration with excellent biological and mechanical properties (Ding et al., 2016; Behera et al., 2017; Farokhi et al., 2018). Furthermore, bone marrow-derived mesenchymal stem cells (BMSCs) embedded in the scaffolds have been widely applied as an advantageous therapeutic option for bone regeneration, and they hold potential to differentiate into osteoblasts both *in vitro* and *in vivo* (Bianco et al., 2001; Crane and Cao, 2014; Oryan et al., 2017).

**FIGURE 1:**
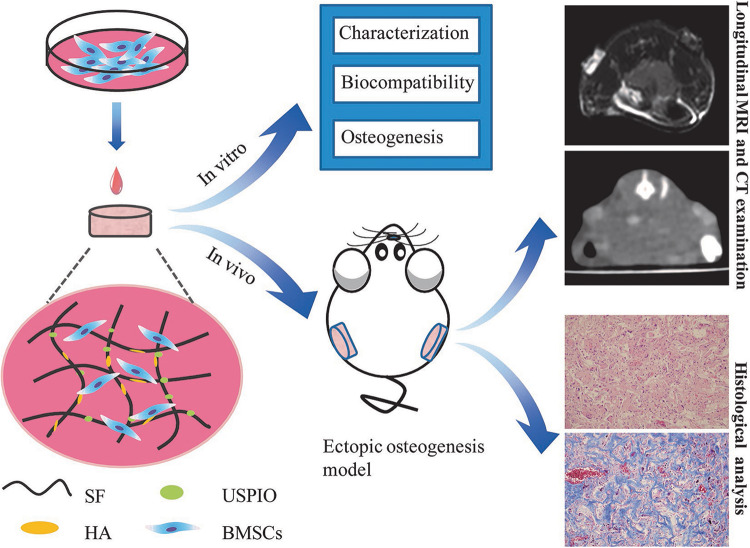
Design of the USPIO-labeled SF/HA multifunctional scaffold system for *in vitro* and *in vivo* studies.

In this study, we incorporated USPIO nanoparticles into SF/HA scaffolds to generate MRI contrast for visualization. After characterization analysis and biocompatibility evaluation, USPIO-labeled and unlabeled scaffolds were cultivated with BMSCs and implanted subcutaneously into the back of nude mice, to monitor scaffolds resorption and bone remodeling at predefined time points by longitudinal MRI (Figure 1). The extent of bone regeneration was further confirmed by CT examination and histological analysis.

## Materials and Methods

### Materials

Bombyx mori silk cocoons in the experiment were kindly donated by Sijia Min from Zhejiang University. Ultrasmall superparamagnetic iron oxide was from Aladdin Reagents Co. Ltd (Shanghai, China), Fetal bovine serum (FBS), alpha modified eagle medium (α-MEM) from Gibco (Grand Island, NY, United States). The Cell Counting Kit-8 was from Dojindo (Kumamoto, Japan) and the LIVE/DEAD^TM^ Cell Imaging Kit was from Invitrogen (Carlsbad, CA, United States). The ALP kit was from Jiancheng Bioengineering Institute (Nanjing, China). TRIzol Reagent, PrimeScript^TM^ RT Master Mix, and SYBR^®^ Premix Ex Taq^TM^ were from Takara Bio (Kyoto, Japan). All other chemicals were purchased from Sigma–Aldrich (St. Louis, MO, United States), and used without further purification. Preparations of 6% SF solution, HA and USPIO nanoparticles are supplied in Supplementary Material.

### Synthesis of 3D Porous USPIO/SF/HA Scaffolds

Fifty mg of HA was dispersed by ultrasonication in 1ml deionized water containing different concentrations of USPIO (0.25, 0.5, 0.75, 1.0, and 1.5%, w/w). Then, 9 ml 6% SF solution was dropped in the mixture of HA and USPIO along with vigorous vortex mixing, and 120 μl of the mixture was pouring in a 96-well plate. Finally, 3D porous scaffolds can be obtained by a freeze-drying process. Scaffolds were sterilized with ^60^Co irradiation before cell seeding.

### Scaffold Characterization

X-ray diffraction (XRD) of HA and USPIO nano-particles was performed using a Rigaku-Rotaflex Diffractometer (RU-200BH) with a Co-ka radiation (*k* = 1.79 Å) at 30 kV and 44 mA. The infrared spectra of the scaffold were determined using an FT-IR spectrometer (Vertex 70, Bruker, Germany). Samples were ground and mixed with KBr at a ratio of 1:5. The data were then recorded at a wave length range of 400-4000 cm^–1^ with the accumulation of 20 scans with a resolution of 4 cm^–1^. The thermal stability was obtained using a Thermo Gravimetric Analyzer (209F3Tarsus, Netzsch, Germany) under N2 atmosphere at a heating rate of 10 °C/min. Mechanical characterization of 3.5 mm height scaffolds was performed by the testing machine (MTS QT/1L, MTS Systems Corporation, United States) at a compression speed of 1 mm/min, and the compressive modulus were then calculated according to the stress-strain data. The microstructure and pore size of the scaffolds was then analyzed by a SEM microscope (XL-30; Philips, Best, Netherlands). For this purpose, scaffolds were fixed in 2.5% glutaraldehyde and coated with a fine layer of gold sputtering. The pore sizes were measured using commercially available software (Image J software, NIH Image, United States). The sample porosity was measured by ethanol displacement method.

### MRI Evaluation *in vitro*

For MRI, the scaffolds were embedded in 1% (w/v) agarose phantoms, and measured with a clinical 3T whole-body MRI scanner (Philips Achieva, Best, Netherlands). T2 weighted imaging (T2WI), T2 mapping, and T2^∗^ mapping sequences were performed. T2WI were acquired using a multi-slice, multi-shot spin-echo sequence [time of repetition (TR) = 3328 ms, time of echo (TE) = 80 ms, field of view (FOV) = 80 mm × 40 mm, matrix size = 64 × 64, and slice thickness = 1 mm]. For transverse (T2) relaxometry, images were acquired at 6 echo times [TE range 8–48 ms] using spin-echo sequences [TR = 1500 ms, FOV = 40 mm × 40 mm, reconstruction matrix = 288, slice thickness = 1 mm]. For transverse (T2^∗^) relaxometry, images at 6 echo times [TE range 5.4–35.1 ms] were acquired by using a multi-shot, multi-slice fast-field gradient-echo sequence [TR = 804 ms, FOV = 40 mm × 40 mm, reconstruction matrix = 112, slice thickness = 0.8 mm, and flip angle = 45°]. T2 and T2^∗^ relaxation times (R2 and R2^∗^) were calculated using the Imalytics Preclinical Software (Philips Technology GmbH, Aachen, Germany).

### Cytotoxicity Assay

Primary isolation of BMSCs and cell passage are supplied in Supplementary Material. For cytotoxicity assays, scaffolds were placed into 48-well plates and seeded with BMSCs at a density of 2.0 × 10^3^ cells/well in advance. Control group without scaffolds was also seeded with the same number of cells. After 1, 3, 5, and 7 days, the culture media was removed and the cell counting kit-8 (CCK-8) solution was added according to the manufacturer’s instructions. The optical density at 450 nm was measured after 2 h of incubation. All experiments were performed in triplicates.

### BMSCs Seeded Onto Scaffolds

Scaffolds pre-treated with basal medium (α-MEM) for 24 h were divided into two groups (USPIO-labeled group and non-labeled group). In preparation, BMSCs at passage 3 were seeded onto scaffolds in 48-well culture plates with 50μl suspension of 2.5 × 10^5^ cells/well. After cell attachment, 500 μl growth medium (α-MEM medium supplemented with 10% (v/v) fetal bovine serum, 1% (v/v) penicillin and streptomycin) or osteogenic medium (with extra addition of 50 μM ascorbic acid 2-phosphate, 10 mM β-glycerol phosphate, and 100 nM dexamethasone) was added. The cell-seeded scaffolds were maintained under standard culture conditions (37°C, 5% CO_2_), changing the culture medium every 3 days.

### Cell Adhesion and Morphology Studies

The morphology of BMSCs on scaffolds was observed by SEM (Hitachi, S-3000N, Japan) after osteogenic induction for 7 and 14 days. The samples were washed with PBS and fixed in 2.5% glutaraldehyde, after which they were dehydrated in graded alcohol, dried in a critical point drier and sputter coated with gold before observation. A Live/Dead assay was performed to assess the growth of BMSCs on both groups. Scaffolds were incubated in calcein (staining for live cells presenting green fluorescence) and ethidium homodimer-1 (staining for dead cells presenting green fluorescence) working solution for 15 min in the dark, and observed by a confocal laser scanning microscope (Olympus FluoView FV10i, Tokyo, Japan). Histological examination was also performed to confirm the existence of BMSCs and USPIO in scaffolds after 7 and 14 days. The specimens were fixed in 4% paraformaldehyde and were dehydrated step-wise using ethanol, immersed in xylene, and embedded in paraffin. Sections of 5 μm were cut and stained by hematoxylin-eosin and Prussian blue, respectively.

### *In vitro* Osteogenic Induction Evaluation

After 1, 7, 14, and 21 days of osteogenic induction, quantitative real-time PCR was performed using the LightCycler^®^480 Real Time PCR System (LightCycler^®^480, Roche, Switzerland). Gene expression of ALP, BMP-2, Collagen I, Runx, and GAPDH (as an endogenous control) were investigated using predesigned primers (supplied in Supplementary Material). Relative expression of the genes was determined using the ΔΔCt method. The alkaline phosphatase (ALP) activity in the medium was assayed using an ALP kit according to the instructions, measuring the absorbance at 405 nm.

### Ectopic Osteogenesis Model

Immune-deficient nude CD-1 nu/nu male mice were purchased from Guangdong Provincial Medical Laboratory Animal Center (China). All animal handling and surgical procedures were performed in accordance with our Institutional Animal Care and Use Committee (Nanfang Hospital, Southern Medical University, China). Groups were showed as follows: non-labeled group, USPIO-labeled group, non-labeled group with BMSCs, USPIO-labeled group with BMSCs. The cell-laden scaffolds were cultured in osteogenic medium for 1 week before implantation. Animals were anesthetized by 0.1% (v/v) of pentobarbital sodium before surgery. Scaffolds were implanted into the bilateral back of the subcutaneous tissue of mice, respectively, and then sutured the skin.

### MRI and CT Evaluation *in vivo*

At 2, 6, and 8 weeks after implantation, mice bearing scaffolds were anesthetized by 0.1% (v/v) of pentobarbital sodium. Then MRI and CT scanning were successively examined. All animals were subjected to T2WI, T2 and T2^∗^ mapping at 2, 6, and 8 weeks after implantation. All sequences were acquired as the *in vitro* MRI experiments described above. Regions of interest (ROI) were manually outlined from the subcutaneous implanted area in the maximal long-axis slice using the Imalytics Preclinical Software (Philips Technology GmbH, Aachen, Germany). The density evolution and new bone formation were assessed using a 256-section multi–detector row CT scanner (Brilliance iCT; Philips Healthcare, Cleveland, OH) with the following parameters: 0.5-s gantry rotation time, 120 kVp tube voltage, 30 mA tube current, 0.67 mm thickness, and 0.2 mm increment. The density of newly formed bone was measured by Philips Brilliance Workspace Versio 3.5 (Philips Medical Systems).

### Histological Examination

After 2, 6, and 8 weeks followed by the MRI and CT examination, one mouse in each group was sacrificed at predefined time points, and the implants were harvested. The specimens were immediately fixed in 4% (wt/v) paraformaldehyde for 48 h, and decalcified in neutral 10% ethylene diamine tetraacetic acid (EDTA) solution for 2 months at room temperature. Then, half of the specimens were dehydrated step-wise using ethanol, immersed in xylene, and embedded in paraffin. Sections of 5 μm were cut and stained by hematoxylin-eosin and Masson trichrome for morphological analysis and bone extracellular matrix deposition. The remaining samples were used to quantify the residual iron oxide particles in scaffolds by inductively coupled plasma mass spectrometry (ICP-MS). After decomposed in digestion system with nitric acid and washed with deionized water, the total amount of iron was detected with high-resolution sector field ICP-MS (Optima 2000DV, Perkin Elmer, United States).

### Statistical Analysis

All data were presented as the mean ± standard deviation of triplicate trials (*n* = 3). Statistical analysis was performed with the SPSS software (version 22.0, IBM, United States). The normality and homogeneity of variance of the data was confirmed by Kolmogorov-Smirnov test and Levene Statistic. Differences in cellular experiment were evaluated with one-way ANOVA (Bonferroni as post-hoc analyses). Repeated-measurement ANOVA was performed to evaluate differences among different time intervals for *in vivo* experiments. Significant differences are given as ^∗^*P* < 0.05, ^∗∗^*P* < 0.01, or ^∗∗∗^*P* < 0.001.

## Results

### Characterizations of SF/HA Composites

**FIGURE 2:**
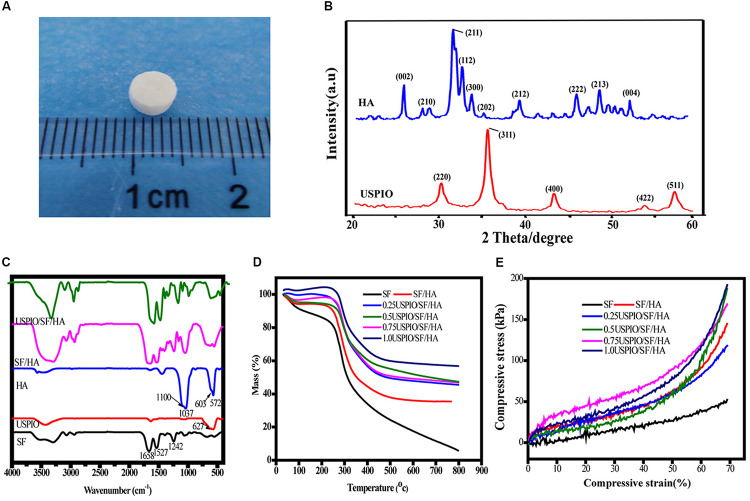
Characterization of scaffolds with different amounts of USPIO incorporated (0–1, w/w%). Gross observation of scaffold **(A)**, X-ray diffraction of HA and USPIO nanoparticles **(B)**, Fourier transform infrared spectroscopy **(C)**, Thermogravimetric analysis **(D)**, and Stress–Strain curve **(E)**.

**FIGURE 3:**
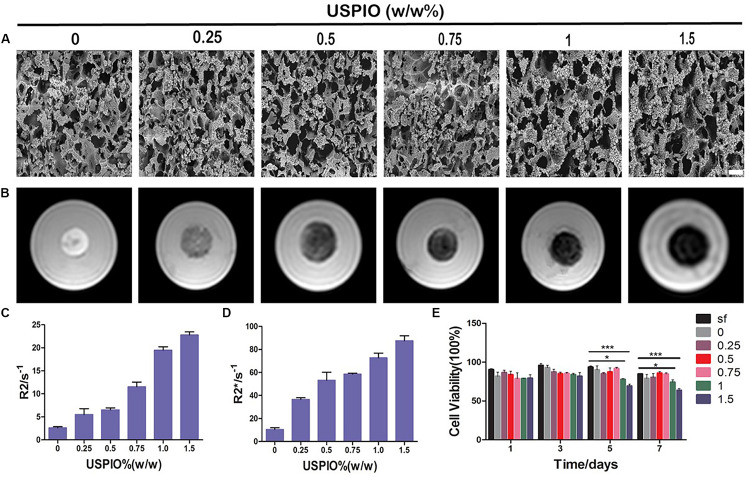
SEM, MRI analysis and cytotoxicity of scaffolds with increasing incorporated amounts of USPIO. **(A)** SEM images show structural properties of scaffolds with incorporation of USPIO nanoparticles. Scale bar indicates 50 μm. **(B)** T2 weighted images demonstrate that the MR signal intensity of USPIO-labeled scaffolds decreased according to USPIO amounts. **(C,D)** Quantitative R2- and R2*-relaxometry analysis. **(E)** CCK-8 results show high cell viability of BMSCs (*n* = 3, *P < 0.05, **P < 0.01, or ***P < 0.001).

As showed in Figure 2A, the scaffold composite was cylinder-shaped with a diameter of 5 mm and height of 3 mm. The XRD of HA and USPIO nano-particles was showed in Figure 2B. The peaks of (2 2 0), (3 1 1), (4 0 0), (4 2 2), and (5 1 1) at 30.1°, 35.4°, 43.1°, 53.6°, and 56.9°confirmed the cubic crystallinity of iron oxide in the form of magnetite (Fe_3_O_4_, Figure 2B). The 2 angles at 26.7°, 31.7°, 46.9°, 49.5° and 53.3° were indexed to be (0 0 2), (2 1 1), (2 2 2), (2 1 3), and (0 0 4) reflections of HA, respectively. The FTIR spectra of scaffolds were separately obtained (Figure 2C). The FTIR spectrum of SF showed amide I, II, and III peaks at 1658, 1527, and 1242 cm^–1^, respectively. The HA spectrum showed the characteristic absorption bands in the region of 1100 cm^–1^, which corresponded to the O–H stretch, and at 603 cm^–1^, which corresponded to the PO_4_^–3^ stretch. The characteristic absorbance peak at 627 cm^–1^ confirmed the Fe–O stretch within USPIO. All the characteristic absorption peaks mentioned above could be found in the composite scaffolds. The mass drop in weight (%) was obtained by thermogravimetry (Figure 2D). The scaffolds with different concentrations of USPIO exhibited better thermal stability than SF alone and simple SF/HA scaffolds. These scaffolds showed almost the same weight loss in the transition temperature of 200–400°C, which indicated that the USPIO probably has positive effects on SF/HA in thermostability. The mechanical properties of the scaffolds are shown in the stress-strain curve (Figure 2E). The compressive modulus of each scaffold was defined by the slope of the initial linear section of the stress-strain curve. The hybrid scaffolds possessed higher compressive modulus (from 0.64 ± 0.08MPa to 1.18 ± 0.13MPa) than pure SF scaffolds (0.61 ± 0.15MPa), and the compressive modulus of the scaffolds containing 0.75% USPIO were the best (1.18 ± 0.13MPa), following by the concentration of 0.25%(1.16 ± 0.10MPa). These results indicated that the incorporation of HA and USPIO nano-particles strengthened the mechanical property and thermal stability of scaffold to a certain extent, which are suitable for further studies. The microstructure of cross sectioned scaffolds was observed by SEM, as presented in Figure 3A. The scaffolds exhibited a porous structure, and the pores were uniform and well interconnected with an average size of 118.4 ± 2.8 μm, presenting a total porosity of 91.5 ± 3.0% (supplied in Supplementary Material). Similar structure was found in the morphology of the USPIO labeled scaffolds, which might be attributed to the low amounts of USPIO incorporated.

### MRI *in vitro*

T2 weighted images of SF and SF/HA scaffolds labeled by different concentrations of USPIO and the corresponding R2 and R2^∗^ values are shown in Figure 3B. The T2WI images revealed that the MRI signal intensity of labeled scaffolds decreased with the increasing concentration of negative contrast agent USPIO. Quantitative R2 and R2^∗^ relaxometry values correlated well with the amount of USPIO incorporated (Figures 3C,D). Strong negative contrast on T2WI images could be observed even with USPIO concentrations lower than 0.5% (w/w). However, incorporation of USPIO concentrations of higher than 1% (w/w) produced apparent MRI image deformation, which made it difficult to measure the boundary and the size of the scaffolds accurately. Therefore, incorporation of USPIO concentration of 0.5–1% (w/w), which produced ideal and uniform contrast enhancement, was considered suitable for visualization of the scaffolds.

### Cytotoxicity Assay

The cell viabilities were assessed by the CCK-8 assay (Figure 3E). The final calculation of the percentage of cell viability was as follows: percentage of cell viability = (*A*_*treatment*_ - *A*_*blank*_)/(*A*_*control*_ - *A*_*blank*_) × 100% (where, *A* = absorbance). There, treatment groups were the cells treated with scaffolds, control groups were with cells contained, and blank groups were only with growth medium contained. Throughout 7 days of culture, no apparent reduction in cell growth rate was found in all scaffolds. However, the viabilities of SF/HA scaffolds with high concentration of USPIO (≥1%, w/w) were slightly lower than those in the non-labeled scaffolds over time (*P* < 0.05), which revealed that the excessive amount of iron particles might affect the growth rate of cells. Therefore, comprehensive considering of the CCK-8 assay, MRI contrast and mechanical property, the SF/HA scaffolds labeled with 0.75% (w/w) USPIO were chosen for the subsequent *in vitro* and *in vivo* experiments.

### Cell Adhesion and Morphology Seeded on Scaffolds

**FIGURE 4:**
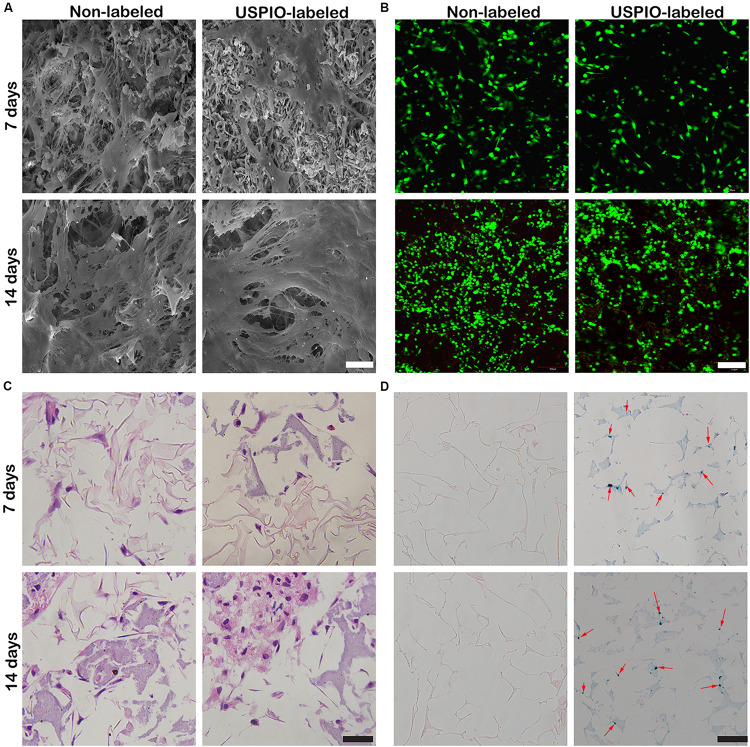
Cell adhesion and growth cultured with USPIO-labeled and unlabeled scaffolds at 7 and 14 days. **(A,B)** SEM and Live/Dead staining show BMSCs adhered to scaffolds. Scale bar indicates 40 μm for SEM and 100 μm for Live/Dead staining. **(C,D)** H&E and Prussian staining of scaffold composites. Scale bar indicates 50 μm.

After 7 and 14 days of culture, the morphology of cells on scaffolds (non-labeled group and 0.75% USPIO-labeled group) was observed by the SEM (Figure 4A). Bone marrow-derived mesenchymal stem cells extended, interconnected on the scaffolds surface in 7 days. After 14 days of culture, multilayer cells overlaid almost the entire surface of the scaffold. The Live/Dead cell analysis (Figure 4B) observed by confocal fluorescence images showed that the vast majority of the cells throughout the scaffolds were stained green (live) with few cells stained red (dead) on the scaffolds. H&E staining also proved the adhesion and growth of BMSCs on the scaffolds by showing an increasing number of cells (Figure 4C). These findings indicated that porous structures of SF/HA scaffold could promote cell attachment and growth. Furthermore, uniform distribution of blue spots stained for iron particles could be observed in USPIO labeled scaffolds by Prussian blue staining (Figure 4D).

### Evaluation of Osteogenic Differentiation

**FIGURE 5:**
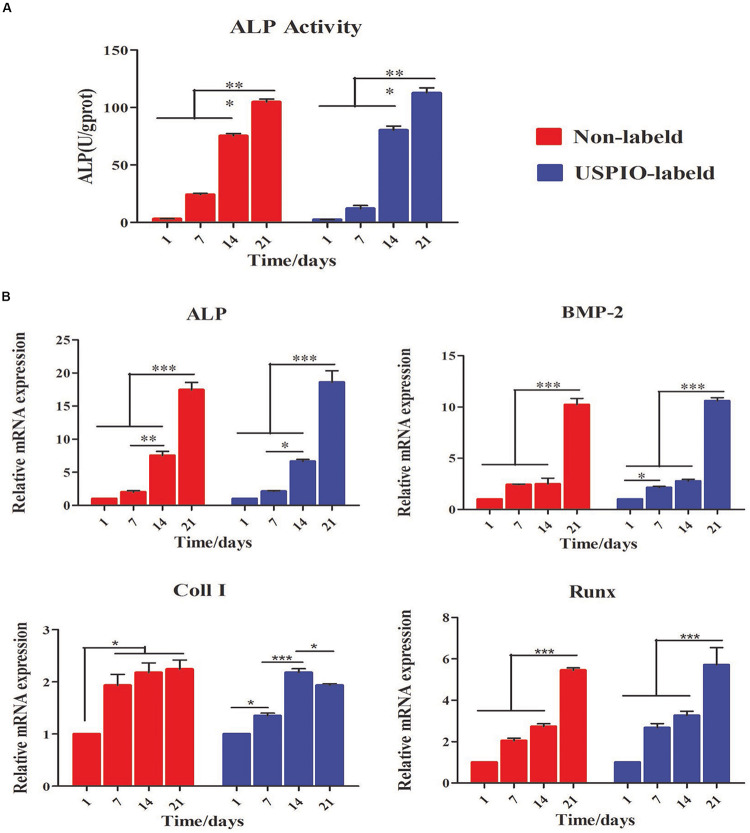
Evaluation of osteogenic differentiation for BMSCs/scaffold composites. **(A)** ALP activity increases for both groups at 1, 7, 14 and 21 days. **(B)** Expression of osteogenic genes (Alp, Bmp-2, Coll I, and Runx) for BMSCs loaded on scaffolds is up-regulated over time (*P < 0.05, **P < 0.01, or ***P < 0.001).

As shown in Figure 5A, the ALP activity, an early marker for osteogenic differentiation, increased significantly in USPIO-labeled and unlabeled scaffolds over time, and reached a peak on day 21 for both groups. Likewise, osteogenic gene expression of BMSCs cultured on both scaffolds was evaluated by qRT-PCR at 1, 7, 14, and 21 days. As illustrated in Figure 5B, the related genes expression of ALP, BMP-2, and Runx demonstrated a significant upregulation on both groups, and reached peak at 21 days. The differences of the ALP, BMP-2, and Runx gene expression between 21 days and other time points were significant (all *P* < 0.001). The expression of Coll I showed a similar tendency, but it reached its peak at 14 days and decreased thereafter. The expression of osteogenic markers demonstrated the osteoconductivity for bone formation of SF/HA scaffolds.

### MRI and CT Evaluations *in vivo*

**FIGURE 6:**
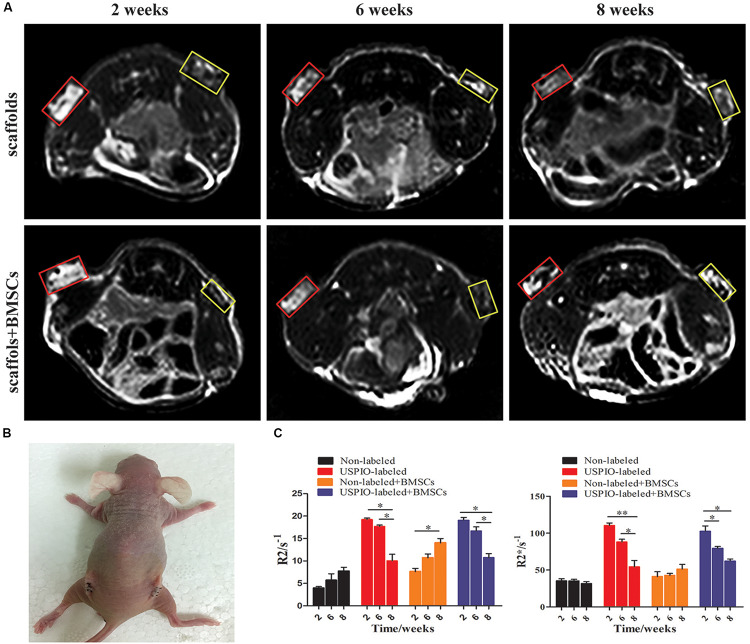
Ectopic osteogenesis model and MR analysis for *in vivo* studies. **(A)** MRI of scaffolds implanted nude mice at 2, 6, 8 weeks (yellow box: USPIO-labeled scaffolds, red box: non-labeled scaffolds). **(B)** Scaffolds implanted on the back of the nude mice bilaterally. **(C)** Quantitative R2- and R2*-relaxometry analysis (*P < 0.05, **P < 0.01, or ***P < 0.001).

The T2WI images of nude mice bearing subcutaneous implants (Figures 6A,B) showed diverse performance in different groups. The signal intensity on T2WI in the USPIO-labeled groups with or without BMSCs decreased from 2 to 6 weeks after implantation, and then increased gradually with the corresponding decrease of the R2 and R2^∗^ values (Figure 6C). For the USPIO labeled groups, the R2 and R2^∗^ values at 8 weeks were significantly lower than those at 2 weeks (both *P* < 0.01). In contrast, signals in the regular implants group showed a tendency to decrease with time, along with the increasing R2 values (*P* < 0.05) and relatively stable R2^∗^ values.

**FIGURE 7:**
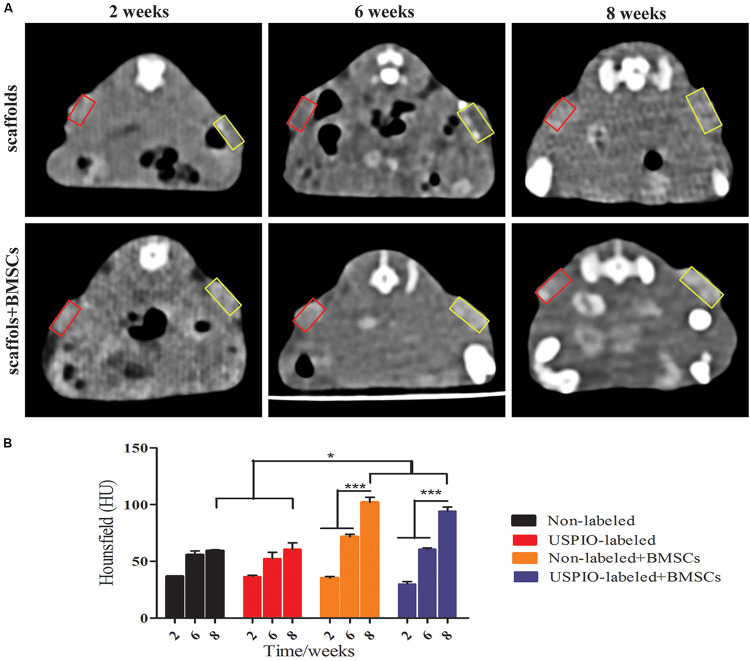
CT analysis for *in vivo* studies. **(A)** CT images of mice show increased density over 2, 6, 8 weeks (yellow box: USPIO-labeled scaffolds, red box: non-labeled scaffolds). **(B)** CT values confirm subcutaneous bone formation of scaffolds (*P < 0.05, **P < 0.01, or ***P < 0.001).

CT imaging was also performed simultaneously to demonstrate the internal changes of implants (Figure 7A). In the BMSCs loaded group, spots of high density on CT were seen at 8 weeks, and the corresponding mean CT value reached 93.9 ± 3.2HU (Figure 7B), which was significantly higher than that at 2 weeks (32.6 ± 1.6HU, *P* < 0.001) and 6 weeks (66.2 ± 5.5 HU, *P* < 0.001). Furthermore, the CT density in the USPIO-labeled groups was comparable to the unlabeled groups at each time point. But higher CT density was observed in the BMSCs loaded group compared with acellular scaffolds (*P* < 0.05), which confirmed the newly formed bone matrix components.

### Histological Examination

**FIGURE 8:**
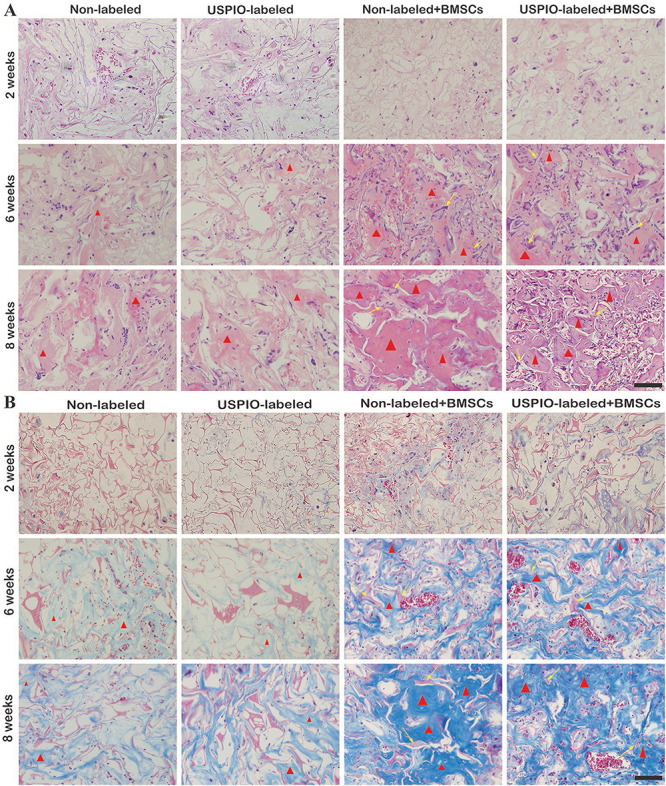
H&E staining **(A)** and Masson trichrome staining **(B)** show osteoid deposition (red triangle) and typical residual scaffold incorporated well within the matrix (yellow arrows) in BMSCs-load scaffolds.

**FIGURE 9:**
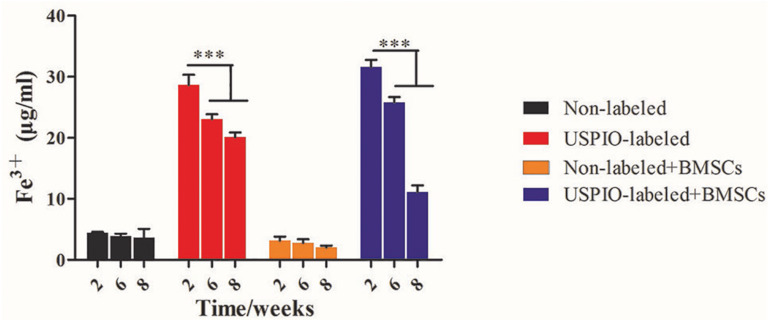
Concentrations of Fe^3+^ by ICP-MS in scaffolds decrease after implantation *in vivo* over time.

H&E and Masson trichrome staining demonstrated that a growing amount of osteoid deposition and neovascularization in scaffolds loaded with BMSCs over 8 weeks. Remnants of the scaffolds were also observed, which was incorporated well within the matrix and decreased over time. In contrast, the acellular scaffolds showed poor osteoid tissue formation (Figures 8A,B). The quantitative results of ICP-MS (Figure 9) proved the degradation of the iron particles over time, which correlated well with the changes of MRI signal *in vivo*.

## Discussion

Bone tissue engineering, which entraps functional cells in 3D implantable scaffolds at the site of injury, has shown promise for the regeneration of bone defects. Recent advances in regenerative medicine have led to new strategies for bone tissue reconstruction (Kim et al., 2014; Melke et al., 2016; Raeisdasteh et al., 2017). Regenerative scaffold-based strategies are proposed to provide structural, biological and biomechanical supports that are imperative for bone regeneration. Sophisticated cell-based techniques are feasible for bone repair, but such approaches are difficult for the proper visualization of the fate of cells and materials after implantation. Our results demonstrated that a 3D SF/HA blended and USPIO labeled scaffold could longitudinally monitor bone tissue engineering by quantitative MRI.

As reported before, the incorporation of nanoparticles could enhance the mechanical property of biomaterials (Dashnyam et al., 2014; Ganesh et al., 2014; Singh et al., 2014b). In this study, we found that SF/HA scaffold is mechanically stronger compared with native silk fibron, which could be attributed to the increased stiffness and compressive strength of native bone by the nano-HA particles (Kasoju and Bora, 2012). Also, the incorporation of USPIO into scaffolds showed better thermal stability than pure SF and SF/HA scaffolds. These results suggest that the scaffolds developed in this study exhibited favorable mechanical property and thermal stability, which are well suited for bone tissue engineering application. Then scaffold composites were confirmed to enhance osteogenic differentiation by facilitating the expression of ALP and osteogenic gene in 21 days *in vitro*. It has been reported that magnetic nanoparticles showed an enhanced osteogenesis to prompt stem cell proliferation and osteogenic differentiation *in vitro* and *in vivo* (Yun et al., 2016; Hu et al., 2018). However, no significant difference was found in osteogenic induction effect between USPIO labeled and unlabeled groups in this study. This may be due to that the low concentration of incorporated USPIO made weak magnetic impact for BMSCs to differentiate.

Thus far, extensive studies have developed scaffolds labeled with various imaging agents in tissue engineering. In this regard, each imaging modality has unique advantages along with intrinsic limitations. For example, Zhang et al. (2016) demonstrated the use of fluorescent labeling coupled to optical imaging for tissue engineering to monitor hyaluronan hydrogels, which was limited by poor light penetration depth of fluorescein. Haralampieva et al. reported scaffolds labeled with radioactive agents used in positron emission tomography imaging toward potential noninvasive tracking of bioengineered muscle tissues (Haralampieva et al., 2016), but it is somewhat limited by poor spatial resolution and radiation risk. Wang et al. utilized CT to noninvasively dynamic monitor biodegradable polymers regarding the microstructure of tissue engineering constructs labeled with gold nanoclusters (Wang et al., 2020). Recent image-guided tissue engineered approaches highlight the superiority of MRI for providing functional information about the biological response of implanted material (Hartman et al., 2002; Szulc and Cheng, 2019). Nevertheless, only few MRI studies have focused on *in vivo* imaging of musculoskeletal tissue implants longitudinally. More recently, we developed a novel multifunctional USPIO labeled cellulose nanocrystal/SF hydrogels that allowed the non-invasive monitoring of hydrogel degradation and cartilage regeneration after *in vivo* implantation in a rabbit model (Chen et al., 2018).

Previous studies have elucidated the relationships between MRI quantitative parameters and regenerative medicine (Cheng et al., 2010; Hu et al., 2018). T2 and T2^∗^ mapping, with the calculated R2 and R2^∗^ values, could track the absorption and function of the scaffolds. R2^∗^ values have been reported to reflect the concentration of USPIO loaded in the scaffolds, thus the increase of R2^∗^ is indicative of the degradation of the USPIO-labeled implants (Chen et al., 2018). However, scaffold degradation and bone regeneration occurred simultaneously within the scaffolds, which may complicate the MRI signals. In this study, the R2 values tended to decrease over time for USPIO-labeled scaffolds. These results hence indicate that the decreased relaxation rate caused by the released iron might outweigh the factor as shown in the non-labeled scaffolds. On the contrary, the R2 values of the unlabeled group with BMSCs increased gradually over time. This can be attributed to the ossification promotion contributed by the BMSCs embedded within the scaffolds. Additionally, density values of CT scanning revealed the evolution of bone generation progress in grafts. Though no remarkable enhancement in density was found on CT values at week 2 and 6 (*P* > 0.05), the increase tendency in MRI R2 signals of BMSCs loaded group was observed. Thus, in our study, the combination of MRI and CT examination provided enough information on both scaffolds degradation and ossification in ectopic bone formation, and this system seemed to be feasible in the follow-up of bone repair. In Prussian blue staining, we found that USPIO particles distributed uniformly in scaffold structures, and the same reduction trends were observed in histological examination and ICP-MS. So we speculate that the iron release progress may reflect the degradation of scaffolds. Although MRI and CT can visualize the multifunctional scaffold and provide valuable feedback on the reconstruction and healing process in depth, one inherent limitation of this method is that an ectopic osteogenesis model was chosen to avoid the bleeding of bone defect in situ influenced on MRI signals. Another main limitation is the lack of measurement of calcification after implantation by quantitative MRI. Future studies are required to employ advanced imaging methods, such as quantitative susceptibility mapping (QSM) and spectral CT, to provide separate quantification of compositions in this multifunctional scaffold system, and to improve comprehensive evaluation of tissue engineering strategies for bone regeneration.

## Conclusion

In conclusion, we developed a USPIO labeled BMSCs-loaded multifunctional scaffold system, which can be feasible for longitudinally monitoring the resorption and function of bone regeneration scaffolds by quantitative MRI and CT. These findings provide new understanding on the non-invasive follow-up of the scaffold system and the potential transitional application of bone tissue engineering strategy for its clinic practice.

## Data Availability Statement

The datasets generated for this study are available on request to the corresponding authors.

## Ethics Statement

The animal study was reviewed and approved by Nanfang Hospital, Southern Medical University, Guangzhou, China.

## Author Contributions

QL designed the study and performed the experiments, analyzed the data, and drafted the manuscript. LF performed the experiments and analyzed the data. ZC involved in the design of study, performed animal experiments, and wrote and reviewed the manuscript. YoL developed scaffold and characterized the properties. YuL and DL contributed to the sample analysis and data interpretation. CY and YX designed the study, discussed the data, wrote and revised the manuscript. All authors contributed to the article and approved the submitted version.

## Conflict of Interest

YoL, YuL, and DL were employed by the company Beogene Biotech. The remaining authors declare that the research was conducted in the absence of any commercial or financial relationships that could be construed as a potential conflict of interest.
