# Supplementary material for: Ultrasmall Superparamagnetic Iron Oxide Labeled Silk Fibroin/Hydroxyapatite Multifunctional Scaffold Loaded With Bone Marrow-Derived Mesenchymal Stem Cells for Bone Regeneration
Source: Front Bioeng Biotechnol. 2020 Jun 30;8:697. doi: 10.3389/fbioe.2020.00697 (PMC7338306; doi:10.3389/fbioe.2020.00697)
Supplement: Supplementary file 1 [file Data_Sheet_1.pdf]

Supplementary Material

1 Supplementary Data

1.1 Preparation of HA

Nano-scaled HA was synthesized by procedures reported previously (Motskin et al.,2009), with slight modifications. Briefly, 0.3M $(NH_4)_2HPO_4$ solution was added into 0.5M $CaCl_2$ solution with controlled speed (12.5 ml/min). This mixture was magnetic stirred for 2 hours at 60℃, followed by adjusting pH to 10 with ammonium hydroxide. After placed at room temperature for 24 hours, the solution was centrifuged at 5000 rpm/min. Then the resulting precipitate was washed with deionized water for 5 times and freeze-dried for 24 hours to generate HA powder.

1.2 Preparation of SF

Briefly, bombyx mori silk cocoons were cut into pieces and boiled for 1 hour with 0.02M Na_2CO_3 added. Then the silk fibroins were washed for several times to remove the sericins. After the degummed silk fibroins were dried at 50℃ for 24 hours, they were dissolved in 9.3M LiBr for 4 hours at 60℃ and dialyzed against distilled water to remove LiBr by using dialysis cassettes (Mw=8-14kDa) for 3 days. The final concentration of the silk fibroin solution was 6% (w/v).

1.3 Preparation of USPIO

Briefly, 1.78g $FeCl_2$ and 1.5g polyethylene glycol were each dissolved by 15 ml deionized water. Then they were blended to get a uniform suspension, followed by addition of 17 ml ammonium hydroxide, 5 ml 3% hydrogen peroxide and 50 ml deionized water. The reaction pH was kept to 11 by using ammonium hydroxide. The mixture was heated at 60℃ for an hour and purified with distilled water. Finally, we obtained USPIO nanoparticles by an external magnetic field.

1.4 Isolation of BMSCs

Four-week-old SD rats were sacrificed with bilateral tibias and femurs harvested under aseptic circumstances. Osteoepiphysis of all bones were removed, and bone marrow was flushed out through a 19-gage needle to obtain single cell suspension. After centrifugation, cells were cultured in α-MEM medium supplemented with 10% (v/v) fetal bovine serum, 1% (v/v) penicillin and streptomycin. The primary BMSCs were cultured in a humidified atmosphere with 5% CO_2 at 37℃ and the medium was changed every 2 days. The cells were passaged after digestion with 0.25% trypsin (Gibco, USA) till they reached 80% confluence. BMSCs cultured to the third passages were used in this study.

2 Supplementary Figures and Tables

2.1 Supplementary Tables

Table 1 Primers for qRT-PCR.

Genes	Forward primer (5'->3')	Reverse primer (5'->3')

Alp	TGCAGGATCGGAACGTCAAT	GAGTTGGTAAGGCAGGGTCC
Bmp-2	CACGAGAATGGACGTGCCC	GCTTCAGGCCAAACATGCTG
Collagen I	AAGGCTCCCCTGGAAGAGA	CAGGATCGGAACCTTCGCTT
Runx	CCAGTTCTGCTCCTCTCCAG	GCCCACAGATTCCTCTTCTG
GAPDH	AGTGCCAGCCTCGTCTCATA	GATGGTGATGGGTTTCCCGT

Table 2 Pore sizes and porosities of SF and SF-based scaffolds.

	Pore diameter (μm) ±SD	Porosity(%)±SD
SF/HA	115.3±1.5	91.7±3.7
USPIO(0.25)/SF/HA	118.6±2.5	90.6±2.6
USPIO(0.5)/SF/HA	120±2.6	92.4±4.1
USPIO(0.75)/SF/HA	116±4.2	93.4±2.5
USPIO(1.0)/SF/HA	123.2±2.9	91.4±3.6
USPIO(1.5)/SF/HA	117.3±3.3	89.8±1.9

REFERENCES

Motskin M, Wright DM, Muller K, et al. (2009). Hydroxyapatite nano and microparticles: correlation of particle properties with cytotoxicity and biostability. *Biomaterials*. 30,3307 - 3317. doi:10.1016/j.biomaterials.2009.02.044
